# From past to future: Digital approaches to success of clinical drug trials for Parkinson's disease

**DOI:** 10.1177/1877718X251330839

**Published:** 2025-04-27

**Authors:** Cen Cong, Madison Milne-Ives, Ananya Ananthakrishnan, Walter Maetzler, Edward Meinert

**Affiliations:** 1 Translational and Clinical Research Institute, Newcastle University, Newcastle, UK; 2 Centre for Health Technology, School of Nursing and Midwifery, University of Plymouth, Plymouth, UK; 3 Department of Neurology, Kiel University, Kiel, Germany; 4 Department of Primary Care and Public Health, Imperial College London, London, UK

**Keywords:** Parkinson's disease, drug discovery, clinical trial, technology

## Abstract

Recent years have seen successes in symptomatic drugs for Parkinson's disease, but the development of treatments for stopping disease progression continues to fail in clinical drug trials, largely due to the lack of clinical efficacy of drugs. This may be related to limited understanding of disease mechanisms, data heterogeneity, poor target screening and candidate selection, challenges in determining optimal dosage levels, reliance on animal models, insufficient patient participation, and lack of drug adherence in trials. Most of the recent applications of digital health technologies and artificial intelligence (AI)-based tools focused mainly on stages before clinical drug trials. Recent applications used AI-based algorithms or models to discover novel targets, inhibitors and indications, recommend drug candidates and drug dosage, and promote remote data collection. This paper reviews the state of the literature and highlights strengths and limitations in digital approaches to drug discovery and development for Parkinson's disease from 2021 to 2024, and offers recommendations for future research and practice for the success of drug clinical trials.

## Plain language summary

In recent years, there have been successes in developing drugs that can help people with Parkinson's disease manage their symptoms but developing drugs that can actually stop the disease from getting worse has been a continuing challenge. Artificial intelligence (AI) has shown promise in improving the success of drug discovery and development for Parkinson's disease. This paper provided an overview of the existing challenges to successful drug trials for Parkinson's disease, recent digital approaches to improve trial success, and remaining challenges that need to be addressed in the future. We found that digital health technologies and AI-based tools were useful for improving the success of clinical drug trials by finding new drug targets and suitable candidates, improving efficiency in pre-clinical evaluation, and facilitating more convenient drug monitoring. While they are helpful in driving innovation and efficiency, drug clinical trials still continue to fail. This may be related to several factors, such as a limited understanding of disease mechanisms, a lack of animal models that can mimic Parkinson's disease and limited real-world patient data for algorithm training. The use of these technologies in clinical drug trials is in its early stages. More research is still needed better to understand the root causes of Parkinson's disease. Researchers are also encouraged to explore how to use existing digital tools to help with drug development at later stages, like large-scale clinical trials and monitoring of drugs after they are approved.

## Background

Over the past 15–20 years, digital health technologies (DHTs) and artificial intelligence (AI) have been applied across various stages of drug research and development, from target discovery to post-market monitoring.^1,2^ DHTs are systems that use computing platforms, connectivity, software, and/or sensors for healthcare and related uses.^3^ These systems can support patient-centered approaches to drug development with improved measurement of disease progression, such as remote data collection.^4,5^ With the power of AI—the ability of algorithms to learn from data and perform tasks automatically without explicit human programming^6^—these systems have offered opportunities to accelerate the drug discovery and development process.^7,8^

The drug development process involves rigorous testing through several phases: Pre-clinical trials assess a candidate's safety in labs and animal models. This is followed by Phase I trials to determine the safety and optimal dosage in small groups of human participants. Phase II then evaluates the drug's efficacy in slightly larger groups of patients. Phase III involves large populations to confirm the drug's effectiveness and safety for regulatory approval. Upon drug approval, the side effects, effectiveness and/or cost will be monitored in Phase IV.^9^ 90% of the drug candidates that passed preclinical evaluation failed during clinical trials and approval.^10^ For neurodegenerative diseases like Parkinson's disease (PD), drug development is even more challenging due to the complexities of the diseases.^7^ PD is a gradually progressive neurodegenerative condition.^10^ It is commonly treated with symptomatic drugs that alleviate the motor and nonmotor symptoms, such as levodopa, dopamine agonists and monoamine oxidase-B inhibitors.^11^ There is still no established disease-modifying treatment that can slow, stop or reverse the progression of PD.^12^ Many attempts were made over the years in this regard, but efforts to translate promising preclinical findings into successful clinical outcomes have continued to fall short.^14–16^

The low success rate in clinical trials of disease-modifying treatments for PD can be attributed to several factors.^10,17^ Preclinical challenges include difficulties in identifying potential drug targets due to limited understanding of the underlying disease mechanisms, lack of sufficient representative in-vitro models (i.e., experiments conducted outside of a living organism) for selecting promising drug candidates,^17^ lack of in vivo models (i.e., experiments conducted within living organisms) that accurately recapitulate the pathology of PD,^18^ as well as issues with determining optimal dosage levels,^19^ and lack of suitable cellular phenotypes for accurate drug screening.^20^ Failures in clinical trials are mainly due to a lack of clinical efficacy and unmanageable toxicity.^10^ Lack of patient participation in trials may also cause delays in obtaining results, small sample size, and shorter duration of experiments, further limiting the generalizability of findings.^21,22^

Previous reviews have presented the potential and challenges in the application of digital technologies and AI for various applications. These include drug discovery in central nervous system diseases,^23^ general drug repurposing/repositioning,^24^ medical devices, pharmaceutical care, and biotechnology,^2^ and diagnosis and management of PD.^25^ Most of them reviewed studies before 2021. In light of the rapid advancements in technologies, this paper offers a review of the current state of technologies, major challenges, and future outlook for drug design and development for PD in this rapidly evolving field. This paper will particularly explore research on DHTs and AI within the past three years. Drawing insights from previous clinical trials, this review will offer recommendations to address limitations and improve study designs for the success of clinical drug trials for PD.

## Digital approaches to drug discovery and development

**Figure 1.**
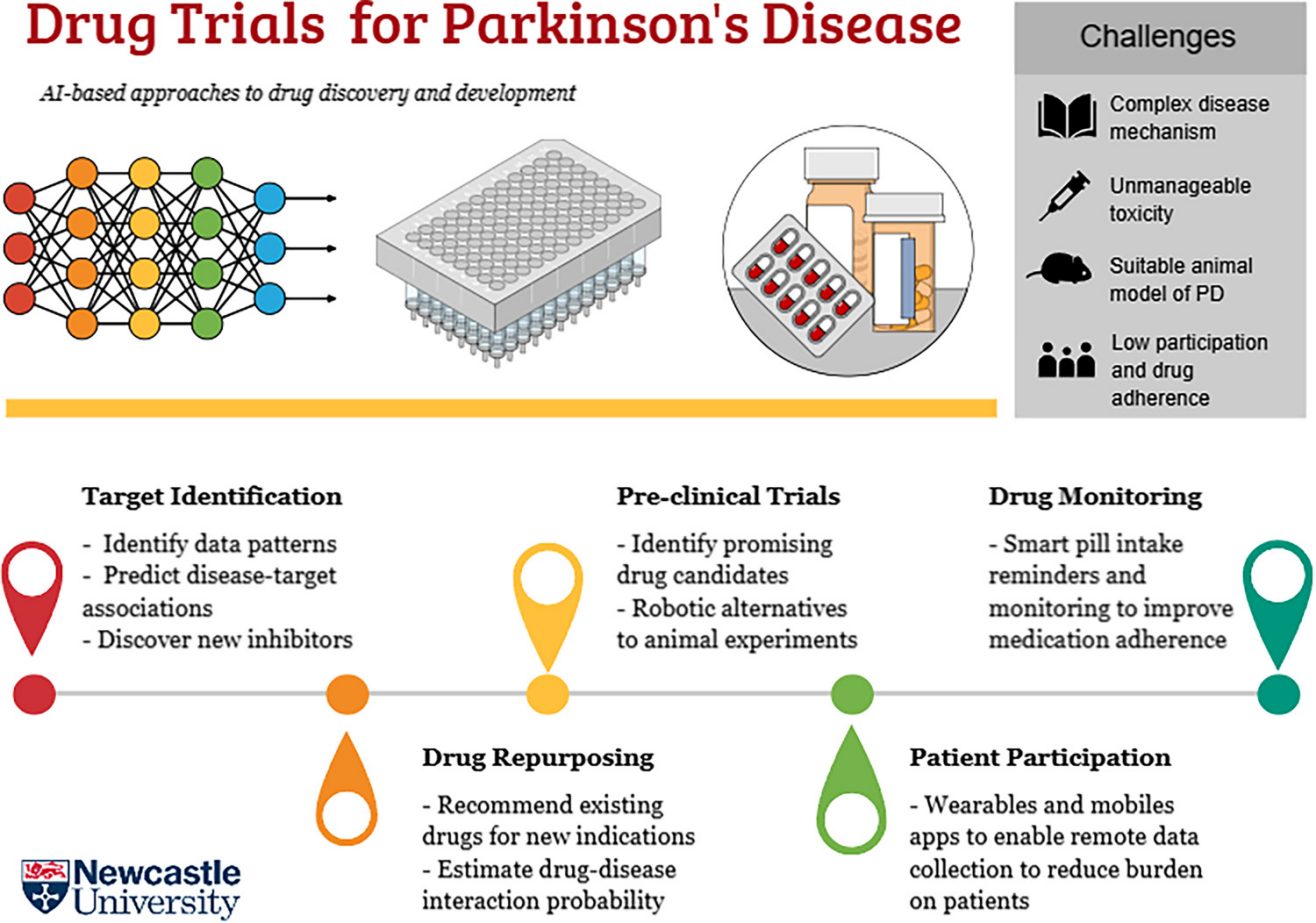
Take-home summary.

Recent years have seen advances in the applications of novel targets and inhibitors, drug repurposing, drug candidate selection, patient participation and drug monitoring for PD (see Table 1 and Figure 1). This section introduces the digital approaches to drug discovery and development in these key areas of applications.

**Table 1.** Summary of failures and digital approaches to drug discovery and development.

Application field	Past failure	Technology used	Contribution	Example
Target identification	Failure to demonstrate effectiveness for indications	Classification algorithms	Identification of patterns in existing patient data to predict disease-target associations and inform personalized treatment	Lin et al., 2023^26^
		Machine learning algorithms like LASSO and mSVM-RFE	Identification of genes associated with immune cell types for PD, enhancing understanding about mechanisms underlying PD and helping discover new inhibitors	Di Stefano et al., 2022^27^
Drug repurposing and candidate selection	Phase III trials (where drug is tested for effectiveness in large groups)	Diffusion algorithms	Recommending potential drugs for repurposing based on similarity to known effective drugs, to improve likelihood of effectiveness	Chen et al., 2021^18^
		Classification algorithms	Identification of associations between existing drugs and diseases, aiding discovery of new repurposing opportunities	Liu et al., 2022^28^
Drug candidate selection	Lack of in vivo animal models that fully mirror PD pathology	Human motor system-based robot-on-a-chips	More accurate representation of human muscle movement than traditional organoids-on-a-chip	Shin et al., 2024^29^
Patient participation and drug monitoring	Non-adherence to drugs during clinical trials	DHTs such as mobile apps and wearable sensors	Monitoring medication adherence in real-time and reminding patients to take the trugs	Christie et al., 2021^30^; Moreau et al., 2023^31^

### Novel targets and inhibitors

A key reason for clinical drug trial failures is lack of efficacy, where drugs fail to demonstrate effectiveness in their indications. AI-based approaches have been widely applied to identify new drug targets and inhibitors, aiming to improve the specificity and efficacy of treatments for PD. A drug target is the biomolecule a drug interacts with (usually specific to a disease), and an inhibitor is the agent that reduces the target's activity, which helps to demonstrate the target's role in disease mechanisms.^33^ By directly targeting disease-specific pathways or molecules, drugs can be more effective in treating the condition. Machine learning methods have been used to support the discovery of drug targets and new inhibitors, such as identifying neuroprotective hexapeptides from walnut protein,^27^ identifying the most important genes associated with immune cell types in the brain for PD,^34^ discovering novel potential inhibitors for cyclin-dependent kinases 5 (Cdk5) through virtual screening,^28^ and identifying potent protein aggregation inhibitors for optimization of in vitro assays.^35^

While AI can help to identify potentially disease-associated genes, the functional validation of these genes remains challenging. For neurodegenerative diseases, such as Alzheimer's disease, it is difficult to validate AI-predicted targets due to the lack of highly reproducible experimental models.^35^ Additionally, AI models often detect correlations rather than causation, leading to the selection of targets that are associated with PD but may not play a direct mechanistic role in disease progression. For example, variants in the Alpha-synuclein (SNCA) gene are the major genes influencing PD, but the association between an SNCA polymorphism and individual outcome still remains unclear.^36^ Likewise, lack of understanding of target engagement also contributed to failures in clinical trials targeting α-synuclein in people with PD.^37^ The k-nearest neighbors algorithm was used to predict drug complexities for PD, but its ability to model complex drug-disease interactions is still limited.^38^

To bridge this gap, AI-based multi-omics analysis can be used to provide insights into the molecular mechanisms underlying PD.^39^ Integrating causal inference models may also facilitate understanding the causal relationship between specific drug targets and disease mechanisms. A recent causal-enhanced method demonstrated higher accuracy in predicting drug–target interaction than traditional AI methods by integrating graph generation and multi-source information fusion.^40^ Causal AI, a predictive AI technology, may also be applied to identify cause-and-effect dependencies between variables in drug discovery.^41^ Such methods may be promising for advancing target discovery for PD.

### Drug repurposing and candidate selection

Drug development is a lengthy and costly process.^23,42^ Drug repurposing has emerged as a promising strategy to accelerate drug development by identifying new indications for existing drugs, saving 3–12 years in development time and cost.^43^ ML-driven knowledge graphs can be used to model the interaction probability between drugs and diseases, facilitating the identification of novel drug candidates and indications.^44^ Diffusion algorithms (e.g., graph diffusion algorithm) were used to recommend potential drugs based on their similarity to known effective drugs or their predicted interactions with disease-related targets.^18^ Classification algorithms have also been used to identify potential indications for existing drugs based on their associations with PD-related proteins (e.g., LProts),^28^ or associating PD genes with Alzheimer's disease.^45^ Natural language processing-based knowledge graph was also used for identifying the most promising drug candidates,^46^ and a combination of deep neural network and ImageNet were adopted for disease modeling and drug screening.^20^

While AI has accelerated the identification of repurposing candidates, AI-recommended drugs still have not seen success in clinical trials. Using IBM Watson, acamprosate, ganaxolone, and lorcaserin were identified as lead candidate drugs with the potential for repurposing to treat L-DOPA-induced dyskinesia, but none ultimately proved successful in *in vivo* studies.^47^ In another example, researchers used an AI-based drug repurposing platform (Standigm Insight™) and identified efavirenz as a modulator of α-synuclein propagation; while it showed potential in animal models, its therapeutic efficacy still requires further investigation.^48^

The failure of AI-driven drug repurposing can be attributed to several factors. One key reason is the discrepancies between animal models and human responses. Animal models are used in vivo models and are paramount to test drug candidates in the stage of preclinical evaluation to assess their safety, efficacy and toxicity before human trials.^49^ Currently, animal models used for testing could not fully recapitulate PD pathology, causing failures in clinical drug trials.^18,44,50^ AI models make predictions based on existing data.^51^ If data are from animal models, this may lead to potential false positives in AI predictions. A breakthrough in this regard is the use of human motor system-based robot-on-a-chips for evaluating drug effects on neurodegenerative diseases, particularly PD.^29^ This advance can contribute to advancement in preclinical drug evaluation, but its benefits beyond preclinical screening are still unclear.

Another big challenge in applying AI algorithms to clinical settings is the limited availability and adoption of real-world, high-quality data (e.g., electronic health records) for model training.^25^ The distribution differences between training data and real-world data (also known as data shifts) could lead to a decline in a clinical AI system's performance.^52^ This may explain why models that perform well in simulations or on curated datasets still fail to generalize to the diverse and complex patient populations encountered in actual clinical trials. Improving AI models’ resistance to data shifts may contribute to narrowing the gap between model training and real-world applications. This could be achieved by deliberately training the model with noisy data and early evaluation of dataset shifts.^53^ Strategies such as synthetic data generation,^54^ transfer learning,^55^ and federated learning^7^ unifying frameworks for dataset shift diagnostics (e.g.,^56,57^) may enhance the generalization of results from one setting to another, so as to improve the clinical translation of AI-powered solutions for PD.

### DHTs for greater patient participation and drug monitoring

In addition to the above issues, obstacles to clinical drug trials for PD also include insufficient patient participation and a lack of high-quality data from diverse patient populations.^58^ These may cause delays in trial completion, enlarge disparities, and limit the generalizability of study results.^21^ Patients with PD often have difficulties in walking and other motor functions, which prevents them from participating in clinical trials in person.

Digital health tools (e.g., mobile apps and wearable sensors) can offer remote monitoring of PD symptoms, thereby promoting patient participation in drug trials.^59,60^ AI-powered wearable sensors, such as Parkinson's KinetiGraph (PKG),^61^ allow continuous tracking of motor symptoms, while smartphone applications like Roche PD Mobile Application v2 collect data on bradykinesia, bradyphrenia, speech, tremor, gait and balance.^62^ DHTs have also been used to address patients’ non-adherence to prescribed drugs during clinical trials, which represents an ongoing challenge to the efficacy evaluation of drugs.^30^ For example, “Smart” pill packs were used to wirelessly track pill usage and send data back to the research team, enhancing drug monitoring and providing data for further drug development.^30^ Another example is Stat-On^TM^, which integrates movement monitoring and drug intake recording to help patients track their symptoms and improve drug adherence.^31^ Moreover, DHTs have the potential to enhance patient recruitment and retention for PD trials through remote or virtual clinical trials. Virtual trials incorporating telemedicine and mobile applications have been found conducive to improving remote participation and retention in clinical trials for PD.^63^ Studies like “AT-HOME PD” have successfully recruited diverse participants across geographic locations and received high participant interest in future remote studies.^64^

As more DHTs are adopted, data comparability issues may arise. Data collected from different systems and tools may not follow the same structure, so it may become challenging to integrate and analyze them, causing data comparability issues. Standardized data collection protocols, data storage, and data analysis are crucial to address this. A metadata framework has been recently proposed to standardize the applications of DHTs in PD trials, enhancing data comparability and supporting regulatory maturity of other drug development tools.^65^ While the framework offers valuable guidance, its implementation remains challenging due to the rapid evolution of digital technologies, various trial designs, and diverse patient populations.

## Conclusions

Digital technologies and AI have shown promise in improving the success of clinical drug trials by having more accurate targets, more suitable candidates, more efficiency in pre-clinical evaluation, and greater drug monitoring.

The current implementation of DHTs and AI-based tools in clinical drug trials is still in the early stages, but early promising results suggest that their integration could lead to great efficiency of drug development for PD, thereby potentially higher chance of success in clinical drug trials. More exploration is needed regarding the underlying mechanisms of neurodegeneration in PD, the use of existing tools in facilitating drug development at later stages (such as Phase II-III clinical trials, sales and post-market monitoring) and the development of AI-powered new models that are tailored for the development of drugs for different levels of PD and different population groups, with high-quality, real-life data. This paper presented an overview of existing challenges to clinical drug trials, recent digital approaches to improve trial success, and remaining challenges to be resolved in future. Due to its limited length, this review did not intend to synthesize all studies related to AI-powered drug discovery and development for PD. A future systematic review is encouraged to compare the performance of relevant AI models, types of data used by the models and their use cases to further understanding of failures in drug trials for PD. Furthermore, the successful implementation of AI in clinical drug trials requires close collaboration between various stakeholders, including clinicians, patients, data scientists, and regulatory authorities. The process of actual collaboration amongst stakeholders and related challenges also requires future exploration. Suggestions in this work have been made to inform studies in this area to continue the success in the development of symptomatic drugs and further the exploration and discovery of disease-modifying treatment for PD, with lessons learned for future success.
